# Editorial: Clinical challenges in pediatric transplant infectious diseases

**DOI:** 10.3389/fped.2024.1425983

**Published:** 2024-07-05

**Authors:** Gabriela Maron, Monica I. Ardura

**Affiliations:** ^1^ Department of Infectious Diseases, St. Jude Children’s Research Hospital, Memphis, TN, United States; ^2^ Department of Pediatrics, University of Tennessee Health Sciences Center, Memphis, TN, United States; ^3^ Department of Pediatrics, The Ohio State University of Medicine, Columbus, OH, United States; ^4^ Pediatric Infectious Diseases and Host Defense Program, Nationwide Children’s Hospital, Columbus, OH, United States

**Keywords:** pediatric, transplant, infections, challenges, cases, solid organ transplantation (SOT), hematopoietic cell transplantation (HCT)

**Editorial on the Research Topic** Clinical challenges in pediatric transplant infectious diseases

The subspecialty of pediatric transplant infectious diseases (pTID) continues to flourish in parallel with advances in solid organ (SOT) and hematopoietic cell transplantation (HCT). The field is ever evolving, with expanding transplant indications as a curative option for malignant and nonmalignant diseases, more children undergoing SOT and HCT, and the increasing and varied landscape of new immunosuppressive agents and emerging immunotherapies, all contributing to a growing number of children who are at risk for a myriad of infectious diseases after transplantation (1–3). In addition, the clinical presentation and severity of the infection may differ depending on the type of immunosuppression. Lastly, new diagnostic technologies and therapeutic agents are emerging but generally lack pediatric-specific data to help guide management, further adding to the complexity clinicians face when caring for this high-risk population.

Pediatric TID is recognized as a key competency within pediatric infectious diseases requiring additional education, and case-based learning has been identified as a useful teaching strategy (4–7). As pTID clinicians and educators, we are constantly learning from the challenging questions we encounter in daily practice and the shared experiences and expertise of colleagues in the field. This special issue of *Frontiers in Pediatrics* is focused on addressing some of the challenges in the diagnosis, treatment, and prevention of infections in children who are transplant recipients by attempting to bridge the gap between clinical practice guidelines that lack inclusion of robust pediatric data and the clinical uncertainty providers may face at the bedside. Providing a patient-centered clinical case for context, leading pTID experts reference available published data, share their reasoning and expertise when approaching frequent clinical dilemmas, and highlight ongoing knowledge gaps in clinical practice that are potential research opportunities.

Given that the differential diagnoses and potential infectious etiologies are quite broad in this population and clinicians rarely know the putative pathogen *a priori* at initial presentation, syndromic topics are introduced including an approach to the transplant recipient with diarrhea, fever, and pulmonary infiltrates, and neurologic manifestations to discern a post-transplant viral meningoencephalitis. Practical perspectives on how to approach suspected donor-derived infections and how to decide whether to proceed or delay transplantation in HCT candidates with viral upper respiratory tract infections are shared. The challenges and evolving management strategies of certain pathogens, including emerging multidrug-resistant Gram-negative bacterial infections, chronic EBV DNAemia in SOT recipients, BK virus-associated hemorrhagic cystitis, and resistant CMV infections in HCT recipients are reviewed. Finally, a practical approach to immunizing SOT candidates and recipients is provided to optimize one of the most effective, yet underutilized, preventative strategies. When there is no one “right answer” to a challenging topic, authors provide a contextual framework from which clinicians can formulate a possible management plan for an individual patient.

As invited editors, it was a privilege to have the opportunity to bring together such an amazing group of junior and “seasoned” leading experts in pTID! We wish to thank all the authors and peer reviewers for their dedication and for sharing their expertise and valuable time in crafting this special pTID issue. We hope readers find the topics educational and useful to their practice. We hope this issue may also serve as an inspirational call to arms to readers who may be medical students, residents, pediatric clinicians, and physician scientists—to join a team of inquisitive, dedicated, and collaborative individuals in the dynamic and exciting subspecialty that is pTID —to work together to move the science forward for our subspecialty and, most importantly, for our patients.
